# EV-D68 neurological disease: tipping the scales toward immunopathogenesis

**DOI:** 10.1172/JCI195839

**Published:** 2025-08-01

**Authors:** Peter W. Krug

**Affiliations:** Vaccine Research Center, National Institute of Allergy and Infectious Diseases, NIH, Bethesda, Maryland, USA.

## Abstract

Over the last decade, there have been multiple outbreaks of enterovirus D68 (EV-D68) disease and associated cases of acute flaccid myelitis (AFM). The underlying cause of EV-D68–induced AFM is contentious; whether spinal cord motor neurons are damaged by direct viral infection, infiltration of immune cells, or a combination of both is not clear. In this issue of the *JCI*, Woods Acevedo and coworkers used a neonatal WT mouse model of EV-D68 infection to attribute paralytic disease to immune cell infiltration into the spinal cord. The results of their work in cytokine-knockout or immune cell–depleted animals effectively argue that immunopathogenesis plays an integral role in EV-D68–induced AFM.

## Overview of EV-D68 disease

Enterovirus D68 (EV-D68) is a respiratory virus that causes severe respiratory infections in children but can, on occasion, enter the central nervous system (CNS) and cause a debilitating paralysis known as acute flaccid myelitis (AFM). Spinal cord anterior horn and brain stem lesions similar to those seen in poliomyelitis are evident by MRI analysis in children presenting with AFM after EV-D68 infection (1). While cerebrospinal fluid (CSF) obtained from affected children around the onset of neurological symptoms has elevated immune cells and viral RNA is routinely detected, there is little to no evidence of infectious virus, possibly due to the presence of EV-D68 antibodies. No treatment has been found to successfully reverse AFM symptoms, indicating that development of rapid diagnostics and intervention prior to CNS involvement is imperative. Understanding virus-host dynamics in and around the sites of motor neuron damage could provide opportunities for intervention to prevent devastating and long-lasting sequelae of infection.

## The source of motor neuron damage

The precise cause of motor neuron damage in EV-D68–induced AFM has been difficult to determine due to many factors, including low case counts, delayed diagnoses, and difficulties with sampling. EV-D68 is cytotoxic, containing three proteases (2A, 3C, and 3CD) that are required for processing structural and nonstructural viral proteins but also cleave host proteins and inhibit cellular functions (2). EV-D68 can infect human induced pluripotent stem cell–derived neurons in vitro, suggesting that cytopathic effects after in vivo infection could disrupt motor neuron function (3). Autopsy samples from early-onset AFM are rare, but viral RNA and protein were present in serial sections of spinal cord from a child who died from an AFM-like illness after EV-D68 infection. CD8^+^ T cell and macrophage infiltration was also noted (4). Perforin colocalized with damaged motor neurons, suggesting that CD8^+^ T cells may have contributed to the patient’s paralysis (5). Taken together, the results from in vitro EV-D68 infection of human neuron cultures and study of a human case implicate both viral cytopathology and immunopathology in the etiology of AFM.

## Neonatal mouse model of EV-D68 paralysis

**Figure 1:**
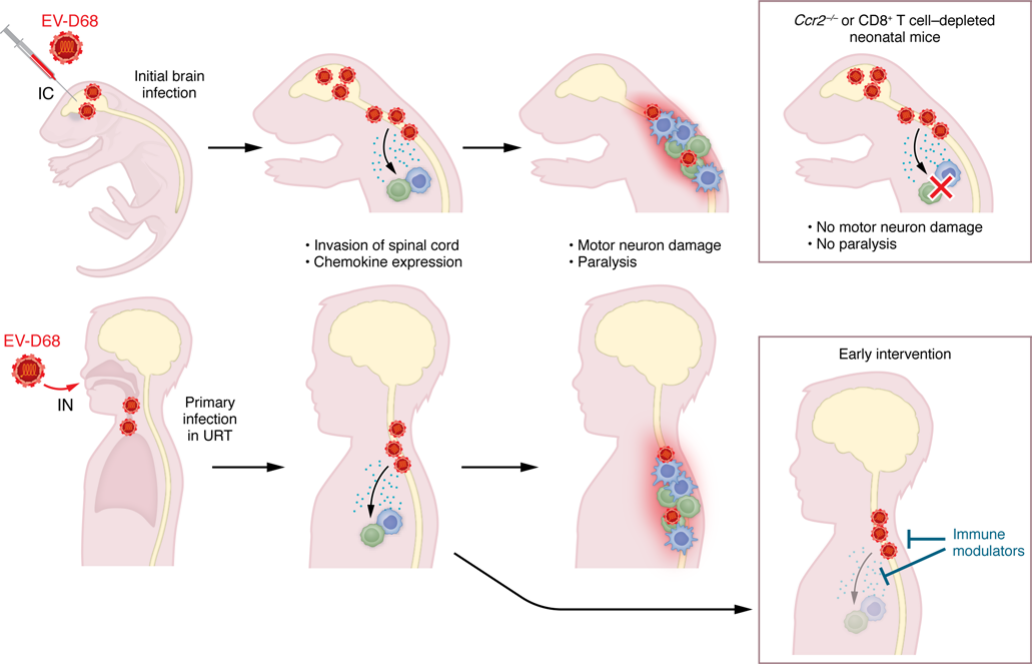
Neonatal WT mouse intracranial infection and CNS disease can parallel EV-D68 infection and progression to AFM. Mice infected with neurovirulent EV-D68 via intracranial (IC) injection undergo viral replication in the brain. In natural infection in children, EV-D68 enters via the intranasal (IN) route and replicates in the upper respiratory tract (URT). In most neonatal mice and infrequently in children, the virus spreads to the spinal cord, causing inflammation and eliciting chemokines that attract and activate T cells and macrophages. Activated T cells and macrophages infiltrate the areas of virus-induced inflammation at the spinal cord, damaging motor neurons and causing limb paralysis. Woods Acevedo et al. (7) reveal that *Ccr2^–/–^* neonatal mice and CD8^+^ T cell–depleted neonatal mice are unable to respond to the inflammation and chemokines elicited by EV-D68 dissemination to the spinal cord, preventing the onset of paralysis. In children, early EV-D68 diagnosis and intervention with specific antiviral and immunomodulatory countermeasures could prevent recruitment of immune cells to the CNS, thereby blocking the onset of AFM.

Animal models recapitulating human EV-D68 disease in mature immunocompetent animals are lacking. Various species of laboratory animals rapidly clear the virus from sites of inoculation, likely due to innate immunity, and the absence of viral replication and dissemination limits their utility for viral pathogenesis studies (6). Woods Avecedo et al. (7) compared intracranial infection of neonatal WT mice with that in two EV-D68 strains: a 2014 B1-subclade virus (termed MO49) shown in some studies to be avirulent (8) and a highly neurovirulent 2014 B2-subclade virus (termed IL52) (9). Both viruses induced cytokines in the spinal cord, but three chemokines were elevated only in IL52-infected mice: CCL2 (also known as MCP-1), CCL7, and CCL12 (MCP-5). IL52 was detected in the CNS and induced paralysis in most infected neonates. Paralysis was diminished in chemokine receptor CCR2-knockout mice, suggesting immune cell infiltrates have a causal role. Further supporting this hypothesis, depletion of CD8^+^ T cells also averted paralytic outcomes. The neonatal mouse pathogenesis after neurovirulent EV-D68 infection largely paralleled the dissemination of virus in humans that progresses to AFM (Figure 1).

## Clinical implications for early detection and treatment

Treatment of AFM patients is mostly supportive, and no countermeasure has been successful in reversing neurologic symptoms, possibly because intervention occurs only after symptom onset. The cytokines found to be upregulated only in neurovirulent EV-D68 infection identified by Woods Acevedo et al. suggest pathways for intervention. While it is not feasible to sample CSF from all children with respiratory infection for these mediators, the neonatal mouse model may reveal blood biomarkers of early EV-D68 dissemination that could be targeted.

In previous studies using this neonatal model, dexamethasone treatment of mice infected with IL52 led to higher viral loads, a worsening of symptoms, and a higher fatality rate (9). Treatments with the anti-enterovirus drug fluoxetine in similar experiments also increased mortality, although they did not decrease viral burden in the tissues. So, while Woods Acevedo et al. demonstrated protection in *Ccr2^–/–^* animals and a possible benefit of treatments that block inflammation and/or immune cell infiltration, in humans a balance may need to be achieved to prevent an increase in viral pathogenesis (Figure 1).

## AFM occurs in a subset of infected children

While infection of neonatal mice is a reliable model of EV-D68–mediated paralytic disease, a wider question is, Are infected neonatal mice a representative model of children with AFM? Woods Acevedo and authors raise the possibility that antigen-independent T cells in newborn mice could play a role in exacerbating disease (7). It is tempting to speculate that T cells of neonatal origin in young children may be involved in AFM development due to their bias toward an innate-like response (10). Developmental differences and variability within the human population may determine the fates of individual human hosts, potentially explaining why some children have EV-D68 infections limited to the upper respiratory tract, while others progress to AFM.

## Concerns (and hope) for the future

EV-D68 continually evolves to evade population immunity, likely allowing spread in the off-peak years of the biennial outbreak pattern (11). The neonatal mouse model could be used to determine the capacity of more contemporary EV-D68 strains to initiate the inflammatory cascade that leads to detrimental T cell infiltration and paralysis. Use of recent outbreak viruses may reveal insight into clinical observations made during the 2022 and 2024 EV-D68 outbreaks, when large numbers of severe respiratory infection cases were unaccompanied with a high incidence of AFM (12). Indeed, in a recent publication (13) by some of the authors of this highlighted article, 2018 B3 subclade viruses exhibited reduced replication in neuronal organoids compared with the 2014 B clade viruses tested. Further, since the B1 and B2 subclade viruses used in Woods Acevedo et al. are no longer circulating, there is some risk that using these older EV-D68 strains to assess virus-specific countermeasures such as antivirals or monoclonal antibodies (14, 15) would overestimate effects, especially when considering contemporary A clade viruses or future B clade viruses.

## Conclusions

Woods Acevedo et al. (7) have presented strong evidence that spinal cord–infiltrating CD8^+^ T cells are capable of causing neuropathogenesis and paralysis following EV-D68 infection. Further investigations into the serum and CSF of infected animals and humans may identify biomarkers that predict progression to neurological symptoms in infected children. Future experiments built on these studies will be uniquely poised to delineate the roles of specific immune cell types in the neuropathogenesis of AFM, enabling the development of effective countermeasures to prevent these devastating infection outcomes.

## Version 1 08/01/2025

Electronic publication
